# Correction: Wang et al. Polycyclic Polyprenylated Acylphloroglucinol Derivatives from *Hypericum acmosepalum*. *Molecules* 2019, *24*, 50

**DOI:** 10.3390/molecules27133986

**Published:** 2022-06-21

**Authors:** Jiao Wang, Mengjiao Shi, Jiajia Wang, Jin Li, Tengfei Ji

**Affiliations:** 1 The Key Laboratory of Plant Stress Biology in Arid Land, College of Life Sciences, Xinjiang Normal University, Urumqi 830054, China; 17364768898@163.com; 2 State Key Laboratory of Bioactive Substance and Function of Natural Medicines, Institute of Materia Medica, Chinese Academy of Medical Sciences & Peking Union Medical College, Beijing 100050, China; 18363030786@163.com (M.S.); wangjiajia@imm.ac.cn (J.W.)

The authors wish to make the following correction to their paper [1]. In the case of hyperacmosin A, the ROESY interaction between H7 and H22 was used to argue that this compound has the 7-exo configuration. Unfortunately, this observation does not support the 7-exo configuration, because H7 has the same chemical shift as one of the two H6s. In hyperacmosin B, the ROESY interaction between H20 and H29 was cited, which would support the 7-endo configuration, not the 7-exo configuration that was assigned.

The Grossman-Jacobs rule described [1] also suggests that hyperacmosins A and B are 7-endo. In 7-exo compounds, the difference in chemical shift between the two H6s is typically 0.3–1.2 ppm, and the chemical shift of C7 is typically 41–44 ppm. In 7-endo compounds, either the difference in chemical shift between the two H6s is 0.0–0.2 ppm, the chemical shift of C7 is 45–49 ppm, or both. Again, both the differences between the chemical shifts of the two H6s (0.08–0.17 ppm) and the chemical shift of C7 (48.1–48.2 ppm) in hyperacmosins A and B support the 7-endo configuration.

The structures of hyperacmosins A and B are shown below: 


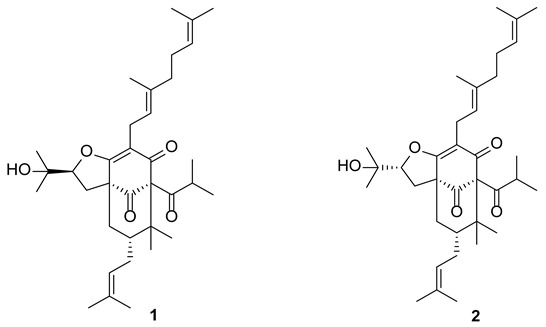


1. The Figure 1 should be changed to:


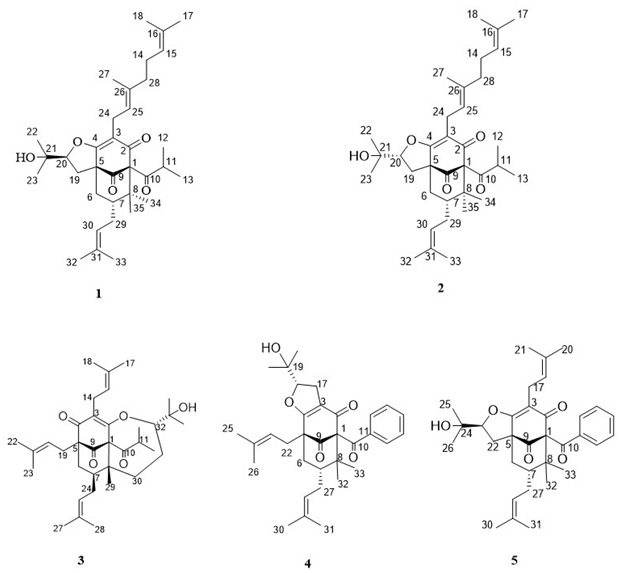


2. The Figure 2 should be changed to:


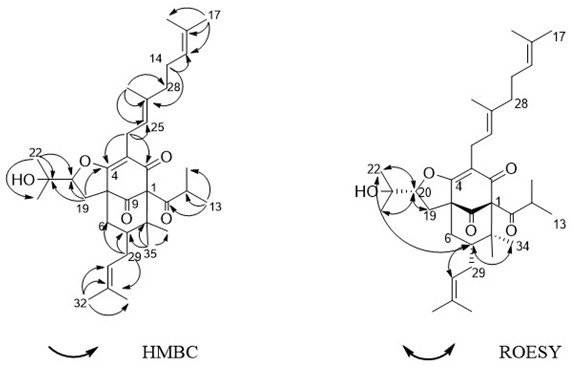


3. The Figure 5 should be changed to:


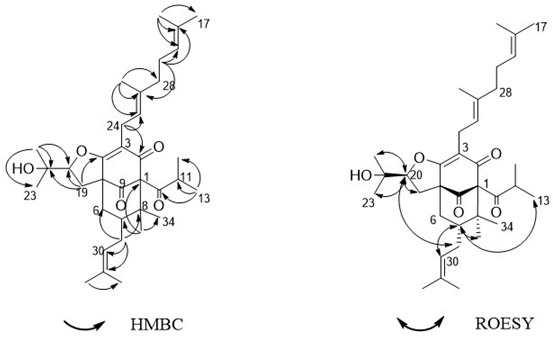


The change does not affect the scientific outcome. The manuscript will be updated and the original will remain online on the article webpage. The authors would like to apologize for any inconvenience caused to the readers by these changes.

## References

[1] Wang J., Shi M., Wang J., Li J., Ji T. (2019). Polycyclic Polyprenylated Acylphloroglucinol Derivatives from *Hypericum acmosepalum*. Molecules.

